# BRPF3-HUWE1-mediated regulation of MYST2 is required for differentiation and cell-cycle progression in embryonic stem cells

**DOI:** 10.1038/s41418-020-0577-1

**Published:** 2020-06-18

**Authors:** Hye In Cho, Min Seong Kim, Jina Lee, Byong Chul Yoo, Kyung Hee Kim, Kwang-Min Choe, Yeun Kyu Jang

**Affiliations:** 1 Department of Systems Biology, College of Life Science and Biotechnology, Yonsei University, Seoul, 03722 Republic of Korea; 2 Initiative for Biological Function & Systems, Yonsei University, Seoul, 03722 Republic of Korea; 3 Colorectal Cancer Branch, Research Institute, National Cancer Center, Goyang, Republic of Korea; 4 Omics Core Laboratory, Research Institute, National Cancer Center, Goyang, Republic of Korea

**Keywords:** Epigenetics, Ubiquitin ligases

## Abstract

Brpf-histone acetyltransferase (HAT) complexes have important roles in embryonic development and regulating differentiation in ESCs. Among Brpf family, Brpf3 is a scaffold protein of Myst2 histone acetyltransferase complex that plays crucial roles in gene regulation, DNA replication, development as well as maintaining pluripotency in embryonic stem cells (ESCs). However, its biological functions in ESCs are not elucidated. In this study, we find out that Brpf3 protein level is critical for Myst2 stability and E3 ligase Huwe1 functions as a novel negative regulator of Myst2 via ubiquitin-mediated degradation. Importantly, Brpf3 plays an antagonistic role in Huwe1-mediated degradation of Myst2, suggesting that protein–protein interaction between Brpf3 and Myst2 is required for retaining Myst2 stability. Further, Brpf3 overexpression causes the aberrant upregulation of Myst2 protein levels which in turn induces the dysregulated cell-cycle progression and also delay of early embryonic development processes such as embryoid-body formation and lineage commitment of mouse ESCs. The Brpf3 overexpression-induced phenotypes can be reverted by Huwe1 overexpression. Together, these results may provide novel insights into understanding the functions of Brpf3 in proper differentiation as well as cell-cycle progression of ESCs via regulation of Myst2 stability by obstructing Huwe1-mediated ubiquitination. In addition, we suggest that this is a useful report which sheds light on the function of an unknown gene in ESC field.

## Introduction

The Brpf family, including Brpf1, Brpf2, and Brpf3, are scaffold proteins of the Myst histone acetyltransferase (HAT) complex; they interact with the Myst HAT, enhancing its acetyltransferase activity and transcriptional activation potential [1], which regulates embryonic development. Brpf1 is necessary to embryonic development in its regulation of *Hox* gene expression [2] as well as hematopoiesis through interaction with MOZ and MORF [3], and is related to the development of some regions in the brain such as the dentate gyrus [4]. Brpf2 is also crucial for embryonic neurodevelopment and fetal erythropoiesis via interaction with Myst2 [5]. Furthermore, the Brpf2/ MOZ complex is required for differentiation induced by retinoic acid in mouse ESCs(mESCs) [6]. However, the function of Brpf3 is relatively unknown.

Histone acetyltransferase Myst2/Hbo1 is important for the maintenance of pluripotency and self-renewal in mESCs. Therefore, Myst2 protein expression must be finely regulated because Myst2 downregulation causes differentiation of mESCs [7]. Myst2 is known to undergo degradation in the regulation of cell proliferation. Fbxw15 degrades Myst2 through Mek1-mediated phosphorylation [8] and CRL4-mediated degradation of Myst2 is induced by ATM/ATR-mediated phosphorylation under UV-damage conditions [9]. Yet, the control mechanism of Myst2 protein expression at the post-translational level in ESCs has not yet been elucidated.

Huwe1, also called ARF-BP1/Mule, is an E3 ubiquitin ligase containing the HECT domain. Huwe1 ubiquitinates N-Myc and the knockout of Huwe1 induces impairment of neuronal differentiation in ESCs. Also, protein expression of Huwe1 increases during differentiation [10], implying that Huwe1 is involved in differentiation. In addition, Huwe1 was previously reported to be involved in DNA replication and DNA damage response [11–14]. However, regulation of pluripotency-related factor such as Myst2 by Huwe1 has not been reported yet.

In the present study, we investigated the function of Brpf3 in mESCs. Our data showed that Brpf3 regulates protein stability of Myst2 by protein–protein interaction. Furthermore, we identified that Huwe1 is a novel ubiquitin ligase of Myst2. Specifically, Huwe1 ubiquitinates Myst2 and this activity was decreased by Brpf3 overexpression, suggesting that Brpf3 blocks the Huwe1-mediated ubiquitination of Myst2. Together, our findings demonstrate for the first time that Brpf3 regulates protein stability of Myst2 by inhibiting Huwe1-mediated degradation and that it is required for differentiation and cell-cycle progression in mESCs.

## Results

### Brpf3 regulates protein stability of Myst2

**Fig. 1:**
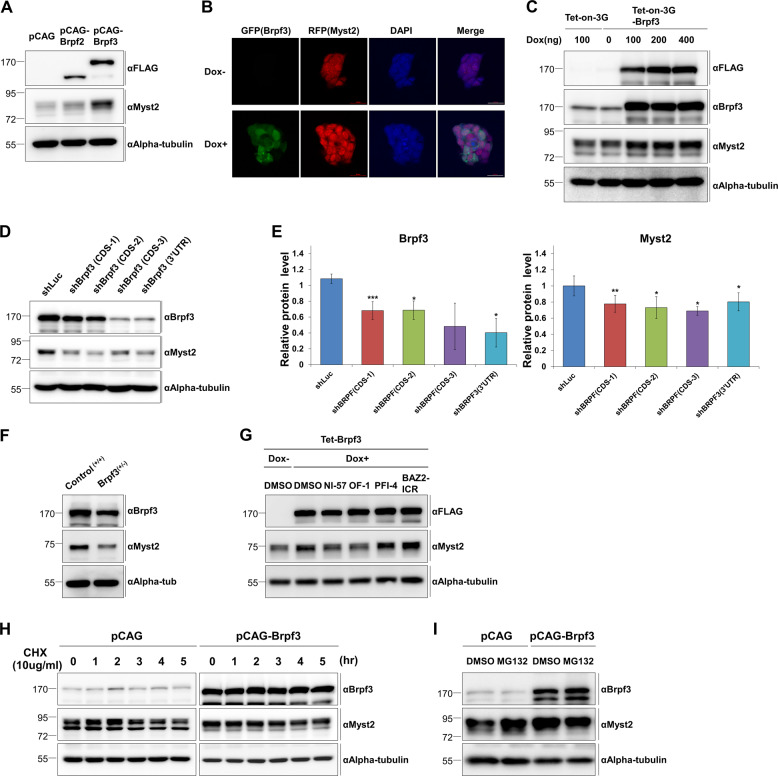
Brpf3 regulates the expression of Myst2 protein. **a** Protein level of Myst2 after overexpression of Brpf2 and Brpf3 was measured by western blot analysis. Alpha-tubulin was used as the loading control. **b** Protein level of Myst2 was confirmed by immunofluorescence staining in inducible Brpf3 overexpression cells using Tet-on-3G system. Overexpression of Brpf3 was induced by treatment with 100 ng of doxycycline. Overexpression of Brpf3 was confirmed by GFP signal and Myst2 was stained with RFP. Scale bar: 20 μm **c** Protein level of Myst2 by inducible overexpression of Brpf3 using Tet-on-3G system was confirmed by western blot analysis. Overexpression of Brpf3 was induced by treatment with indicated amounts of doxycycline. Alpha-tubulin was used as the loading control. **d** Protein expression of Myst2 was analyzed by western blot in control (shLuc) and *Brpf3* knockdown cell lines (shBRPF3 CDS-1, CDS-2, CDS-3, 3’UTR). Alpha-tubulin was used as the loading control. **e** Quantification of results from **d**. Protein levels were normalized to that of alpha-tubulin. The expression level of control (shLuc) was set at 1. (*n* = 3). *p < 0.05, **p < 0.01, ***p < 0.001, compared with control. **f** Protein expression of Myst2 was analyzed by western blot in control (normal E14tg2A) and Brpf3 haploinsufficient mESCs. Alpha-tubulin was used as the loading control. **g** Protein level of Myst2 was determined by western blot in Brpf3-inducible overexpression cells treated with BRD domain inhibitors (Ni-57, OF-1, PFI-4, and BAZ2-ICR). Overexpression of Brpf3 was induced by treatment with 100 ng of doxycycline. Alpha-tubulin was used as the loading control. **h** Protein level of Myst2 in Brpf3-overexpressing cells after treatment with 10 μg/mL cycloheximide for indicated times was investigated by western blot analysis. Alpha-tubulin was used as the loading control. **i** Protein level of Myst2 in Brpf3-overexpressing cells after treatment with 10 μM MG132 for 6 h was analyzed by western blot. Alpha-tubulin was used as the loading control.

To investigate the function of Brpf3 in ESCs, we constructed mESC E14tg2A (E14) cells stably expressing FLAG-tagged Brpf3 and Brpf2. The expression of Myst2 protein was increased in Brpf3-overexpressing cells but not in Brpf2-overexpressing cells (Fig. 1a). The data suggest that Myst2 protein is induced by Brpf3-overexpression but not by its homolog Brpf2. To test whether the upregulation of Myst2 protein observed in Brpf3-overexpressing cells is the result of off-target effects, we examined the effect of inducible overexpression of Brpf3 using the Tet-on/off system on the increased expression of Myst2 protein. Brpf3 overexpression by doxycycline treatment induced the increase of immunofluorescent Myst2 signal, concomitant with the Brpf3 signal (Fig. 1b). Consistent with our immunostaining data, the Myst2 protein level was increased by induction of Brpf3 overexpression as confirmed by western blot analysis (Fig. 1c), but Myst2 mRNA levels were not changed (Supplementary Fig. S1a). Next, we investigated whether *Brpf3* deficiency affects Myst2 expression using western blot and RT-qPCR analyses in both shRNA-based knockdown (*KD*) cell lines and *Brpf3* haploinsufficient mESCs created by CRISPR-CAS9 (Supplementary Fig. S1b, c). Our data revealed that Myst2 protein levels were significantly decreased in *Brpf3*-deficient ESCs relative to control *KD* cells (Fig. 1d, e) as well as in *Brpf3* haploinsufficient mESCs (Fig. 1f). Besides, the increased protein levels of Myst2 by Brpf3 overexpression were reverted by Brpf3 inhibitors Ni-57 and OF-1, but not by Brpf1 inhibitor PFI-4 and BAZ2B protein inhibitor BAZ2-ICR as used for negative control (Fig. 1g), suggesting that Brpf3 regulates Myst2 protein stability.

### Brpf3 negatively regulates proteasomal degradation of Myst2

Regulation of Myst2 protein expression is important in ESCs, but degradation of Myst2 in ESCs has not been thoroughly studied. We examined whether Myst2 is degraded in ESCs by using cycloheximide and MG132 treatment. First, the protein levels of Myst2 in mESCs were gradually reduced in a time-dependent manner by treatment with cycloheximide (Supplementary Fig. S2a). In addition, the accumulation of Myst2 protein was observed in ESCs treated with MG132, a proteasomal inhibitor (Supplementary Fig. S2b). Moreover, our in vivo ubiquitination assay revealed that Myst2 is ubiquitinated in mESCs (Supplementary Fig. S2c), suggesting that Myst2 is eliminated by ubiquitin-mediated proteasomal degradation in mESCs. Since our data indicate that Brpf3 was involved in the regulation of Myst2 protein levels, we further investigated whether overexpression of Brpf3 affects Myst2 protein stability in mESCs. The degradation of Myst2 was inhibited by Brpf3 overexpression in the presence of cycloheximide (Fig. 1h). The inhibitory effect of Myst2 protein degradation caused by MG132 treatment was not significant in Brpf3-overexpressing cells compared with that in control ESCs (Fig. 1i). Collectively, our data suggest that Myst2 is proteasomally degraded in mESCs and its protein stability may be controlled by the presence of Brpf3.

### Huwe1 forms complex with both Brpf3 and Myst2 and is a novel ubiquitin E3 ligase of Myst2

**Fig. 2:**
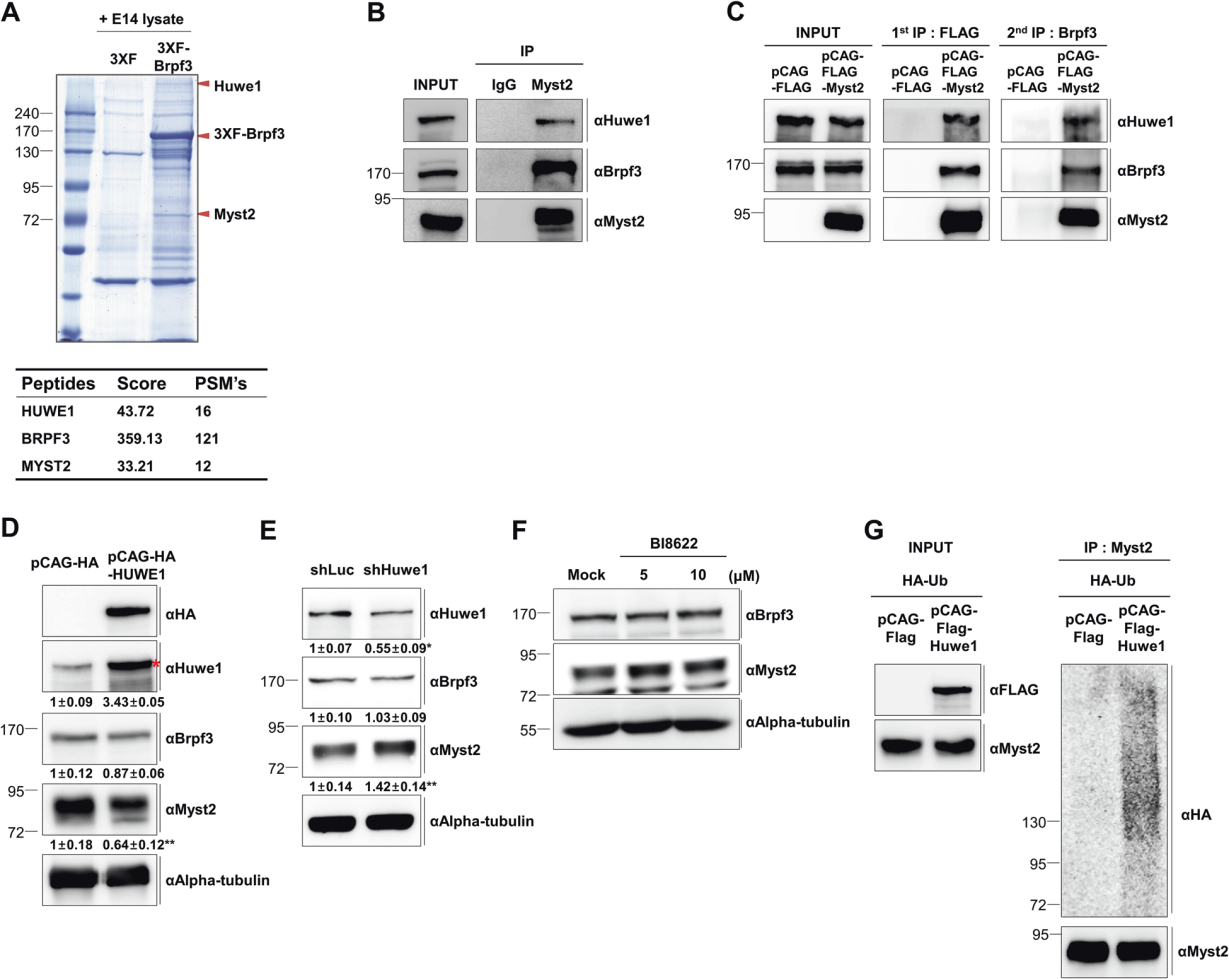
Huwe1 forms complex with both Brpf3 and Myst2 and ubiquitinates Myst2. **a** Coomassie blue staining of proteins associated with Brpf3. Specific Brpf3-interacting proteins were purified by immunoprecipitation using anti-FLAG affinity gel and identified by MS analysis. Identified proteins are indicated on the right. **b** Interaction of the Huwe1-Brpf3-Myst2 complex was analyzed by co-immunoprecipitation using Myst2 antibody. Co-immunoprecipitation was performed in MG132 treated mESCs. Immunoprecipitates were analyzed by western blot with anti-Myst2, anti-Brpf3, and Huwe1 antibodies. **c** Simultaneous interaction between Huwe1, Brpf3, and Myst2 was confirmed by sequential immunoprecipitation. In first immunoprecipitation, interaction was determined using anti-FLAG affinity gel, and then following second immunoprecipitation was carried out with eluted immunoprecipitates from first immunoprecipitation using Brpf3 antibody. **d** Protein expression of Brpf3 and Myst2 in Huwe1-overexpressing cells were investigated by western blot analysis. E14tg2a cells were transfected with plasmids expressing pCAG-HA (empty vector) and pCAG-HA-Huwe1. Alpha-tubulin was used as the loading control. *p < 0.05, **p < 0.01, ***p < 0.001, compared with control. **e** Protein expression of Brpf3 and Myst2 in Huwe1 depleted cells were investigated by western blot analysis. Alpha-tubulin was used as the loading control. *p < 0.05, **p < 0.01, ***p < 0.001, compared with control. **f** Protein expression of Myst2 in Huwe1 inhibited mESCs was analyzed by western blot using Huwe1 inhibitor (BI8622). Indicated amounts of BI8622 was treated for 24 h to inhibit Huwe1. Alpha-tubulin was used as the loading control. **g** Ubiquitination of Myst2 in Huwe1-overexpressing cells was analyzed by immunoprecipitation. pCAG-FLAG and pCAG-FLAG-Huwe1 were co-transfected with HA-tagged Ubiquitin in 293T cells and treated with 10 μM MG132 for 6 h. Protein lysates were immunoprecipitated with anti-Myst2 antibody and detected by western blot with anti-FLAG, anti-Myst2, and anti-HA antibodies.

Since Brpf3 disturbed proteasomal degradation of Myst2, in order to identify which ubiquitin ligase binds and ubiquitinates Myst2 in mESCs, we performed mass spectrometry (MS) analysis using total protein extracts of mESCs and gel-affinity-purified FLAG-Brpf3 expressed in 293T cells. Our proteomic data revealed that Brpf3 can interact with Huwe1 ubiquitin ligase (Fig. 2a). We then verified the interaction between Brpf3, Myst2, and Huwe1 by co-immunoprecipitation endogenously in mESCs (Fig. 2b) and ectopically in 293T cells (Supplementary Fig. S3). Further, to identify whether these proteins form a Huwe1-Brpf3-Myst2 complex, we carried out sequential immunoprecipitation (Fig. 2c). At first immunoprecipitation, the interaction of Myst2 with Brpf3 and Huwe1 was confirmed, and at following second immunoprecipitation, Brpf3 was detected for its interaction with Huwe1. These results showed that Myst2, Brpf3, and Huwe1 interact with each other simultaneously, suggesting that Huwe1 is a potential ubiquitin ligase for Myst2 in mESCs. We also examined whether the overexpression of Huwe1 affects protein stability and ubiquitination of Myst2. Our data indicate that overexpression of Huwe1 caused downregulation of Myst2 protein levels while *Huwe1* deficiency by shRNA-based knockdown caused upregulation of Myst2 protein levels (Fig. 2d, e). Similar to result from *Huwe1* knockdown, the inhibition of Huwe1 by Huwe1-inhibitor BI8622 induced upregulation of Myst2 protein levels (Fig. 2f). Further, overexpression of Huwe1 caused downregulation of Myst2 protein levels as well as an increase in Myst2 ubiquitination (Fig. 2g). Thus, these data suggest that Huwe1 is a novel ubiquitin ligase of Myst2.

### Brpf3 inhibits degradation of Myst2 by protein–protein interaction

Next, we hypothesized that Brpf3 prevents degradation of Myst2 by interaction because Brpf3 is a scaffold protein that interacts with Myst2 HAT and improves enzymatic activities [1]. An elegant study revealed that the N-terminal domain of Brpf3 is important for interaction with Myst2 [15]. Based on this previous study, we constructed four different deletion mutants (N127, ΔN127, ΔPWWP, and PWWP) for investigating the Brpf3 domain required for inhibition of Myst2 degradation (Supplementary Fig. S4a). Our co-immunoprecipitation data confirmed that the N-terminal region of Brpf3 is critical for interaction with Myst2 (Supplementary Fig. S4b). Next, we determined whether the overexpression of Brpf3 deletion mutants affected Myst2 protein stability. Our data showed that overexpression of the Brpf3 deletion mutant containing only an N-terminal 127aa (N127) increased the Myst2 protein level, while overexpression of the Brpf3-ΔN127 mutant had no effect on the Myst2 protein level (Supplementary Fig. S4c). In addition, we tested whether Myst2 protein expression is affected by the PWWP domain (PWWP), known to recognize the H3K36 methylation or PWWP deletion mutant (ΔPWWP). Our data indicate that the Myst2 protein level was only marginally changed in mESCs overexpressing only the PWWP domain, but the Myst2 protein level was considerably increased in cells expressing the ΔPWWP mutant (Supplementary Fig. S4d), suggesting that the PWWP domain was not necessary for the regulation of Myst2 protein stability.

### Huwe1-mediated degradation of Myst2 is disturbed by Brpf3

**Fig. 3:**
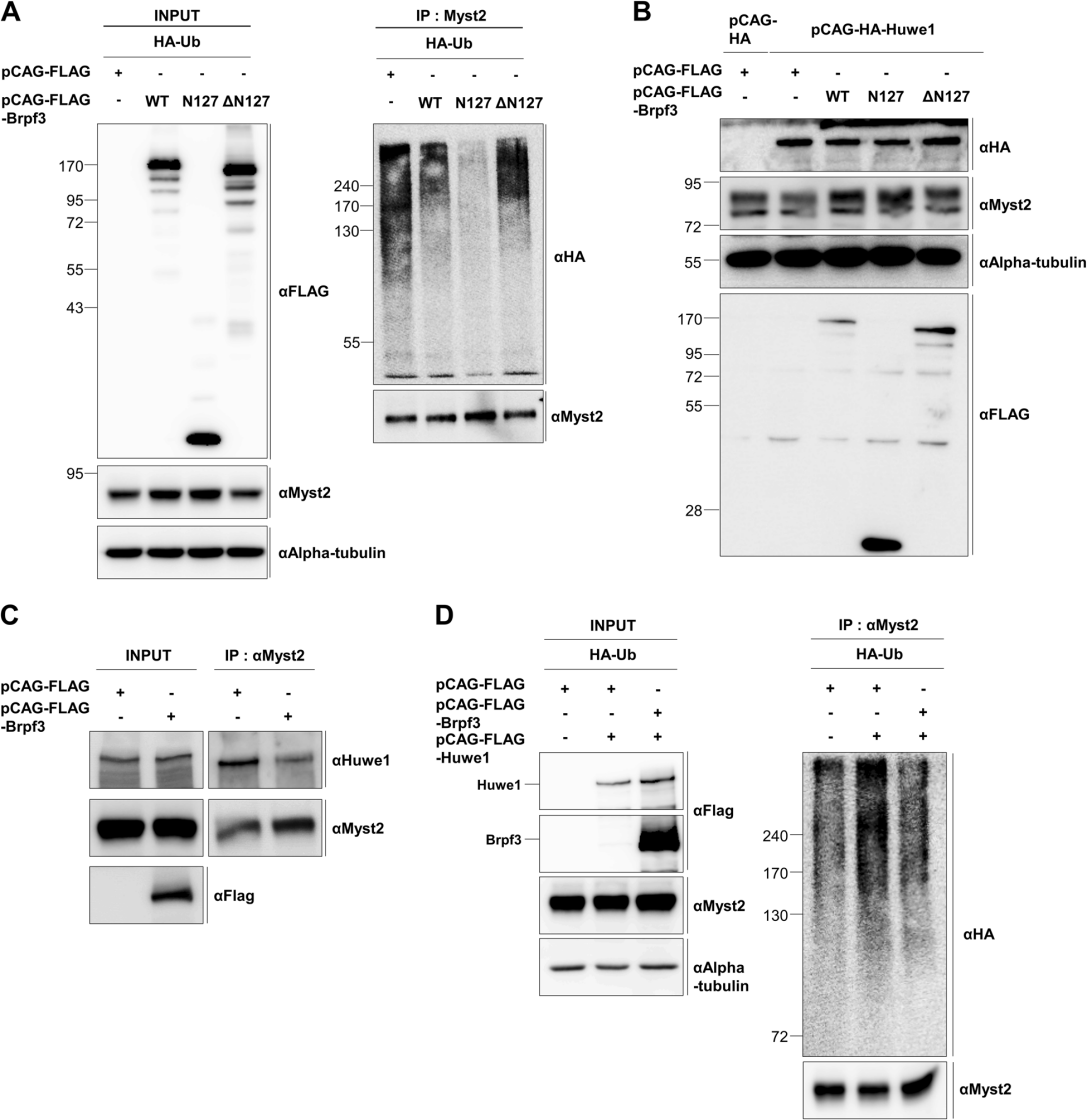
Protein stability of Myst2 is regulated by interaction with Brpf3. **a** Ubiquitination of Myst2 in Brpf3 WT-, N127-, and ΔN127-overexpressing cells was analyzed by immunoprecipitation. pCAG-FLAG and pCAG-FLAG-Brpf3 (WT, N127, ΔN127) were co-transfected with HA-tagged Ubiquitin in 293T cells and treated with 10 μM MG132 for 6 h. Protein lysates were immunoprecipitated with anti-Myst2 antibody and detected by western blot analysis with anti-FLAG and anti-Myst2 antibodies. Alpha-tubulin was used as the loading control. **b** Protein expression of Myst2 in Huwe1-overexpressing cells with Brpf3 overexpression was analyzed by western blot. pCAG-FLAG and pCAG-FLAG-Brpf3 (WT, N127, ΔN127) were co-transfected with pCAG-HA-Huwe1 in E14tg2a cells. Alpha-tubulin was used as the loading control. **c** Interaction of Huwe1 with Myst2 in Brpf3-overexpressing cells was investigated by immunoprecipitation. Protein lysates were immunoprecipitated by anti-Myst2 antibody and were analyzed by western blot with anti-FLAG, anti-Huwe1, and anti-Myst2 antibodies. **d** Ubiquitination of Myst2 in Brpf3 and Huwe1-overexpressing cells was analyzed by immunoprecipitation. pCAG-FLAG and pCAG-FLAG-Brpf3 were co-transfected with pCAG-FLAG-Huwe1 in 293T cells and treated with 10 μM of MG132 for 6 h. Protein lysates were immunoprecipitated with anti-Myst2 antibody and detected by western blot with anti-FLAG, anti-Myst2, and anti-HA antibodies.

We next investigated whether the enhanced protein stability of Myst2 by the overexpression of Brpf3 involves attenuation of Myst2 ubiquitination. As expected, our data revealed that ubiquitination of Myst2 was attenuated by overexpression of Brpf3 wild type (WT) as well as its N-terminal part (N127) compared with that of the empty vector (Fig. 3a). On the contrary, a notable decrease in ubiquitination was not observed in the deletion mutant of Brpf3 lacking its N-terminal part (ΔN127) (Fig. 3a), suggesting that physical interaction between Brpf3 and Myst2 may contribute to the attenuation of the ubiquitination-mediated degradation of Myst2. As our data suggest that Brpf3 inhibits the degradation of Myst2 by interaction, we attempted to investigate whether Brpf3 blocks Huwe1-mediated ubiquitination of Myst2. To examine the interrelationship between Brpf3 and Huwe1, HA-tagged Huwe1 was co-transfected with FLAG-tagged Brpf3 (WT as well as N127 and ΔN127 mutants) and Myst2 protein expression was examined. Consistent with our previous data, the protein level of Myst2 was decreased by the overexpression of Huwe1. However, the Myst2 protein level was restored by overexpression of Brpf3 WT or the N127 mutant despite Huwe1 overexpression, but not by the Brpf3-ΔN127 mutant (Fig. 3b). To figure out the correlation between the Brpf3 domain and its interaction with Huwe1, we performed co-immunoprecipitation using FLAG-tagged Brpf3 (WT as well as N127 and ΔN127 mutants). Both the Brpf3-N127 and Brpf3-ΔN127 mutants interacted with Huwe1, but the interaction of Huwe1 with Brpf3-N127 was more distinct than that of Brpf3-ΔN127 (Supplementary Fig. S5), implying that the N-terminal domain of Brpf3 is important for the interaction with Huwe1 as well as the interaction with Myst2. We further tested whether Brpf3 overexpression influences the interaction between Myst2 and Huwe1 using co-immunoprecipitation. Our data show that overexpression of Brpf3 weakened the interaction between Myst2 and Huwe1 (Fig. 3c). Furthermore, we determined that the Huwe1-mediated ubiquitination of Myst2 was abated by the overexpression of Brpf3 (Fig. 3d). Collectively, we propose that Brpf3 disturbs the interaction between Myst2 and Huwe1, thereby inhibiting the Huwe1-mediated ubiquitination of Myst2.

### The K155 and K576 residues of Myst2 are potential ubiquitination acceptor sites

**Fig. 4:**
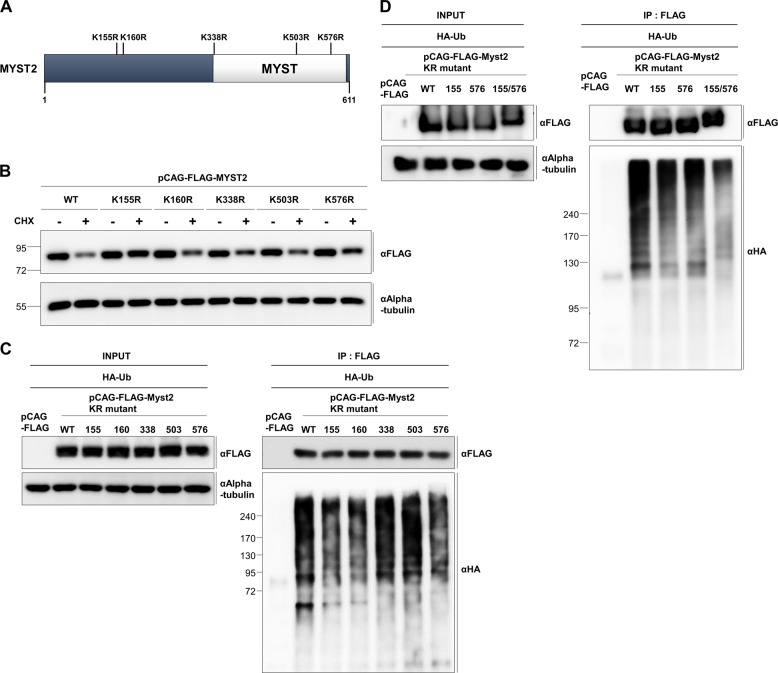
Identification of an ubiquitination acceptor site within Myst2. **a** Schematic representation of predicted ubiquitination acceptor site within Myst2. **b** Ubiquitination of Myst2-wild type (WT) or single lysine-mutated form (K155R, K160R, K338R, K503R and K576R) was analyzed by immunoprecipitation. pCAG-FLAG and pCAG-FLAG-Myst2 WT or KR mutant were co-transfected with HA-tagged Ubiquitin. After treatment with 10 μM MG132 for 6 h, protein lysates were immunoprecipitated using anti-FLAG affinity gel and detected by western blot analysis with anti-FLAG and anti-HA antibodies. **c** Protein level of Myst2-WT and single lysine-mutated form after treatment with 10 μg/mL cycloheximide for indicated times was investigated by western blot analysis. Alpha-tubulin was used as the loading control. **d** Ubiquitination of Myst2-wild type (WT), single lysine-mutated form (K155R and K576R) and double lysine-mutated form (K155/576R) were analyzed by immunoprecipitation. pCAG-FLAG and pCAG-FLAG-Myst2 WT or KR mutant were co-transfected with HA-tagged Ubiquitin. After treatment with 10 μM MG132 for 6  h, protein lysates were immunoprecipitated using anti-FLAG affinity gel and detected by western blot analysis with anti-FLAG and anti-HA antibodies.

We next constructed Myst2 mutants containing single point mutations of lysine residues which is predicted ubiquitination sites to identify the ubiquitination sites of Myst2 in mESCs (Fig. 4a). We first examined the effect of these point mutations on protein stability of Myst2 after cycloheximide treatment. Our data revealed that the half-lives of the K155R and K576R mutants were moderately increased compared with that of wild-type Myst2 after cycloheximide treatment, but unchanged in other mutants (Fig. 4b). To determine the ubiquitination pattern of Myst2 KR mutant, we performed an in vivo ubiquitination assay using FLAG-tagged Myst2 mutants and HA-tagged ubiquitin. Ubiquitination of Myst2 K155R, K160R, and K576R mutants was markedly reduced compared with that of wild type Myst2 (Fig. 4c). In addition, we found that ubiquitination of K155 and K576 simultaneous mutated Myst2 (K155/576R) was highly decreased compared with wild type Myst2 and single mutated Myst2 (K155R and K576R) (Fig. 4d). Together, our data suggest that K155 and K576 residues are potential ubiquitin acceptor sites of Myst2 ubiquitinated by Huwe1 in mESCs.

### Myst2 protein is degraded during differentiation

**Fig. 5:**
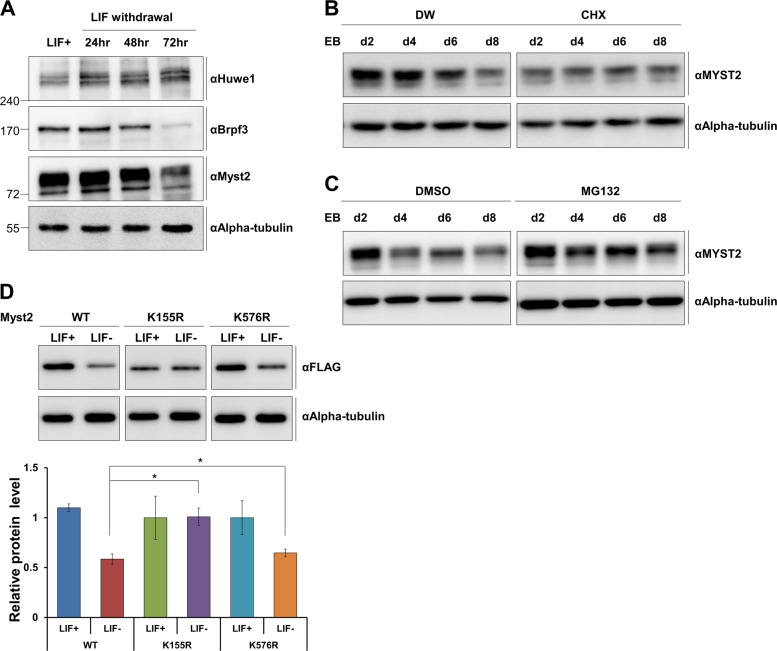
Protein level of Myst2 is decreased by proteasomal degradation during differentiation. **a** Protein expression of Huwe1, Brpf3, and Myst2 during LIF-withdrawal-induced differentiation was analyzed by western blot. Alpha-tubulin was used as the loading control. **b** Protein expression of Myst2 after treatment with 10 μg/mL cycloheximide for 5 h during EB differentiation was investigated by western blot analysis. Alpha-tubulin was used as the loading control. **c** Protein level of Myst2 in Brpf3-overexpressing cells after treatment with 10 μM MG132 for 6 h during EB differentiation was analyzed by western blot. Alpha-tubulin was used as the loading control. **d** Protein level of Myst2-WT, K155R, and K576R during differentiation in Myst2-WT, K155R, and K576R stably expressed mESCs was analyzed by western blot. Differentiation was induced by withdrawal of LIF for 2days. Alpha-tubulin was used as loading control (upper panel). Quantification of results from western blot was normalized to that of alpha-tubulin. The expression level of control (Myst2-WT, LIF+) was set as 1. (*n* = 3). *p < 0.05, **p < 0.01, ***p < 0.001, compared with Myst2-WT (LIF-) (lower panel).

To determine the roles of Huwe1, Brpf3, and Myst2 in pluripotency and the differentiation of mESCs, we measured the protein expression pattern of Huwe1, Brpf3, and Myst2 during differentiation. Myst2 and Brpf3 protein levels were decreased during differentiation while Huwe1 levels were increased (Fig. 5a), consistent with previous reports [7, 10]. These results suggest that the reduction of Myst2 protein stability during differentiation may occur via the attenuation of Brpf3-mediated Myst2 protein expression and Huwe1-mediated ubiquitination, implying the antagonistic role of Brpf3 compared with Huwe1 in mESCs. Next, we assessed the degradation pattern of Myst2 protein during differentiation. The reduction in Myst2 protein levels was markedly accelerated by treatment with cycloheximide during differentiation (Fig. 5b). Moreover, the steady-state level of Myst2 protein became rather accumulated upon treatment with MG132 despite the induction of differentiation (Fig. 5c). Furthermore, the K155R and K576R mutations were able to reduce the degradation of Myst2 protein during differentiation compared with that of wild-type Myst2 (Fig. 5d), suggesting that Myst2 protein is degraded by proteasomal action during differentiation.

### Regulation of Myst2 protein stability is important for proper differentiation in mESCs

**Fig. 6:**
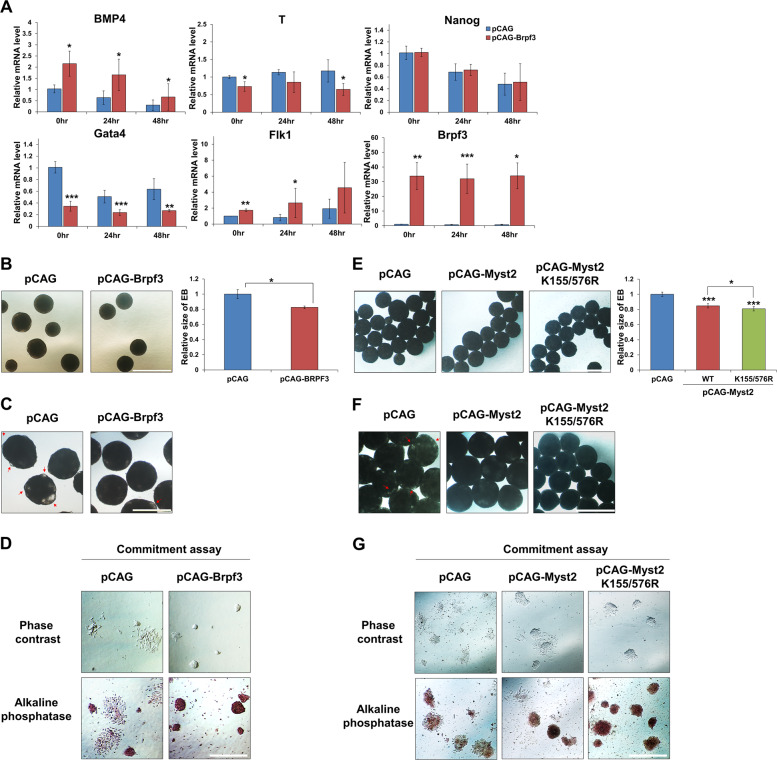
Regulation of Myst2 protein by Brpf3-Huwe1 is required for proper differentiation in mESCs. **a** mRNA expression level of Bmp4, T, Gata4, Flk1, and Nanog during differentiation was analyzed by RT-qPCR in control (pCAG) and Brpf3-overexpressed mESCs (pCAG-Brpf3). Differentiation was induced by withdrawal of LIF for indicated times. mRNA expression levels were normalized to that of GAPDH. The expression levels of the control were set at 1. (*n* = 3). *p < 0.05, **p < 0.01, ***p < 0.001, compared with control. **b** Embryoid-body (EB) formation ability in control (pCAG) and Brpf3-overexpressing mESCs (pCAG-Brpf3) was investigated using the hanging drop assay. Phase-contrast images of EB at two-days after EB formation (left panel) and quantification of the size of EBs (*n* = 3; right panel). More than 30 EBs were measured for each independent experiment. The size of EB control was set at 1. *p < 0.05, **p < 0.01, ***p < 0.001, compared with control. Scale bar: 1 mm. **c** Cyst formation in EB was observed in control (pCAG) and Brpf3-overexpressing mESCs (pCAG-Brpf3). Phase-contrast images of EB at 6-days after EB formation. Red arrows indicate cystic structure of EB. Scale bar: 1 mm. **d** Morphology of control (pCAG) and Brpf3-overexpressing mESCs (pCAG-Brpf3) after induction of differentiation by LIF withdrawal was investigated by phase-contrast images and alkaline phosphatase staining. Scale bar: 0.5 mm. **e** Embryoid-body (EB) formation ability in control (pCAG) and Myst2 wild-type- or ubiquitination defective mutant (K155/576 R)- overexpressing mESCs was investigated using the hanging drop assay. Phase-contrast images of EB at two-days after EB formation (left panel) and quantification of the size of EBs (*n* = 3; right panel). More than 30 EBs were measured for each independent experiment. The size of EB control was set at 1. *p < 0.05, **p < 0.01, ***p < 0.001, compared with control. Scale bar: 1 mm. **f** Cyst formation in EB was observed in control (pCAG) and Myst2 wild-type- or K155/576 R mutant- overexpressing mESCs. Phase-contrast images of EB at six-days after EB formation. Red arrows indicate cystic structure of EB. Scale bar: 1 mm. **g** Morphology of control (pCAG) and Myst2 wild type or K155/576 R mutant overexpressing mESCs after induction of differentiation by LIF withdrawal was investigated by phase-contrast images and alkaline phosphatase staining. Scale bar: 0.5 mm.

We further investigated the effects of Myst2 dysregulation by Brpf3 overexpression on differentiation of mESCs. Our previous study revealed that depletion of Myst2 caused reduction in Bmp4 and Flk1 levels and elevation in T and Gata4 levels [7]. By contrast, our RT-qPCR analysis demonstrated that Brpf3 overexpression resulted in upregulation of Bmp4 and Flk1 and downregulation of T and Gata4, suggesting that overexpression of Brpf3 has antagonistic effects on the depletion of Myst2 (Fig. 6a). Since the dysregulation of pluripotency- and differentiation-related genes in Brpf3-overexpressing cells might cause defects in differentiation process, we investigated whether ectopic expression of Brpf3 influenced embryoid bodies (EBs) formation, which is a common method to assess the differentiation, by observing EB morphology. We found that Brpf3 overexpression decreased the size of EBs (Fig. 6b) and the cyst formation within EBs was abnormally delayed (Fig. 6c). To elucidate whether the overexpression of Brpf3 promotes resistance to differentiation cues, we performed a lineage commitment assay as previously described [16]. The induced differentiation of control cells proceeded normally without any notable delays in the culture supplemented with LIF. This was confirmed by morphological changes such as a diffuse epithelial appearance (Fig. 6d). However, Brpf3-overexpressing cells exhibited rounded and compact colonies characteristic of undifferentiated ESCs (Fig. 6d). Consistent with the morphological changes, a high intensity of AP staining was observed in Brpf3-overexpressing cells compared with the control cells (Fig. 6d). Since Myst2 K155R and K576R mutants were resistant from proteasomal degradation during differentiation (Fig. 5d), we sought to determine whether K155/576R mutation of Myst2 affected differentiation. The size of EB derived from Myst2 K155/576R-overexpressing cells was slightly but significantly decreased compared with that from wild-type Myst2-overexpressing cells (Fig. 6e). In addition, the cyst formation within EBs was dramatically delayed by Myst2 K155/576R mutant overexpression, compared with the control (Fig. 6f), while *Myst2* deficiency induced increase of the EB size and cyst formation (Supplementary Fig. S6a, b). Furthermore, Myst2 K155/576R-overexpressing cells showed rounded and compact morphologies as well as higher intensity of AP relatively to wild-type Myst2-overexpressing cells (Fig. 6g), whereas depletion of Myst2 caused flattened and diffused colonies with low intensity of AP (Supplementary Fig. S6c). Together, we conclude that Brpf3- and Huwe1-mediated regulation of Myst2 during differentiation is required for proper differentiation in mESCs.

### Regulation of Myst2 by Huwe1-Brpf3 is involved in cellular response during replication stress and cell-cycle progression

**Fig. 7:**
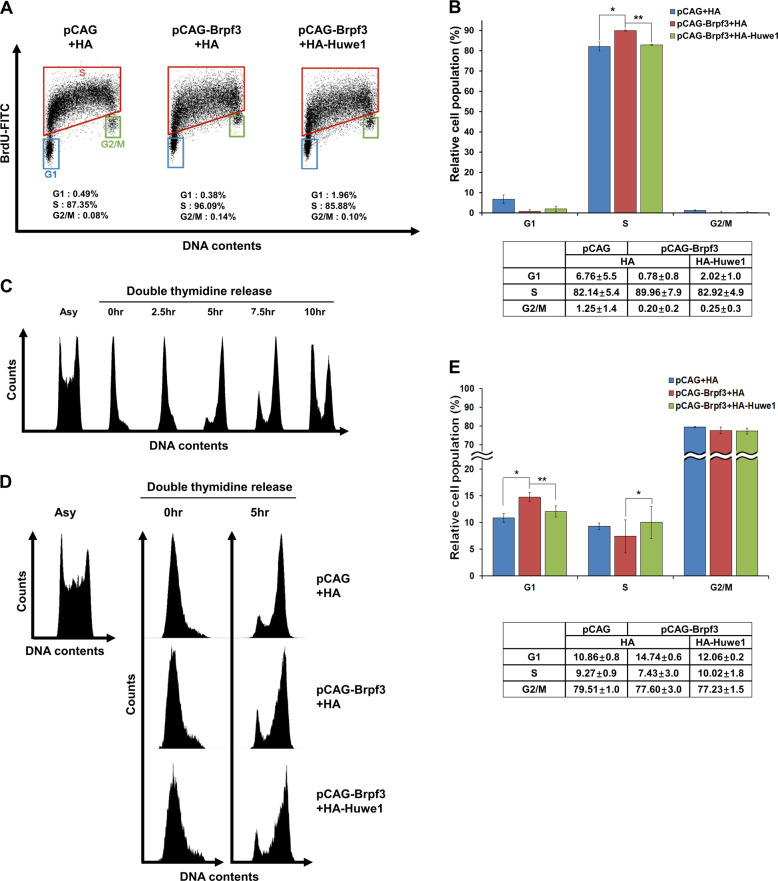
Regulation of Myst2 protein by Brpf3-Huwe1 partnership is involved in both DNA damage response and cell-cycle progression in mESCs. **a** Representative dot blot of BrdU incorporation and DNA content (PI) in Brpf3-overexpressed mESCs and Brpf3-Huwe1- overexpressed mESCs. The percentage of cells in the different cell-cycle phases is indicated. **b** Bar graph representing relative cell populations in cell-cycle phases G1, S, and G_2_/M. *p < 0.05, **p < 0.01, ***p < 0.001, compared with control cells. **c** Cell-cycle progression was analyzed by FACS. Asy means asynchronous cells. For G1-phase synchronization, thymidine was treated for two times and after releasing progression, cell-cycle progression at indicated time was detected. **d** Representative histogram of cell-cycle distribution in Brpf3- overexpressed mESCs and Brpf3-Huwe1-overexpressed mESCs after double thymidine block and release. **e** Bar graph representing relative cell populations in cell-cycle phases G1, S, and G_2_/M. *p < 0.05, **p < 0.01, ***p < 0.001, compared with control cells.

Cell-cycle progression and differentiation are functionally linked in mESCs. Since we confirmed the regulation of differentiation through control of Myst2 stability by Brpf3 and Huwe1, we investigated the regulation of cell-cycle progression. It is previously reported that depletion of *Brpf3* mitigates response after replication stress [15]. Then, we tested whether the effect of Brpf3 on replication stress is rescued by Huwe1. When Brpf3 was overexpressed, BrdU-positive S-phase cells were slightly but significantly increased and it was reverted by Huwe1 overexpression (Fig. 7a, b), suggesting the role of Huwe1-Brpf3 interaction in response of replication stress. In addition, because Myst2 accelerates cell proliferation [17], we checked the function of Myst2 on cell-cycle progression in mESCs. The transition from G1 phase to S phase was blocked by treatment with double thymidine and then the cell-cycle progression was released as previously described [18] (Fig. 7c). Myst2 overexpression promoted cell-cycle progression compared with control (Supplementary Fig. S7a), while Myst2 depletion by shRNA-based knockdown slowed down the cell-cycle progression (Supplementary Fig. S7b). Then, we investigated that Huwe1-Brpf3 partnership controls cell-cycle progression by regulating Myst2 stability. While Brpf3-overexpressing cells progressed faster through the cell-cycle as compared with control cells after releasing cell-cycle for 5-h, Huwe1 overexpression alleviates early progression of cell-cycle in Brpf3-overexpressing cells (Fig. 7d, e). Thus, it might be safer to say that regulation of Myst2 by interaction between Huwe1 and Brpf3 is also involved in cell-cycle progression as well as differentiation of mESCs.

## Discussion

The present study provides novel insight into understanding the role of Brpf3 in mESCs, while identifying Huwe1 as a novel ubiquitin E3 ligase that targets Myst2. Myst2 is degraded by Huwe1-mediated ubiquitination during the differentiation of mESCs and Brpf3 regulates Myst2 protein stability by interfering with Huwe1 through protein–protein interaction.

A recent study revealed that *Brpf3*-knockout mice did not show remarkable change of phenotype for mouse development and survival [19]. However, our results showed that Brpf3 regulates protein stability of Myst2 and blocks Huwe1-mediated ubiquitination of Myst2. More specifically, our data indicated that only the Brpf3 mutant containing the N-terminal 127 residues (N127) interacts with Myst2, enhancing protein stability (Fig. 3). In addition, the Brpf3-N127 truncated mutant is more favorable for interaction with Huwe1 rather than the Brpf3-N127 deletion mutant (ΔN127) (Supplementary Fig. S4). Because N-terminal 579 residues of Brpf3 remained in spite of *Brpf3-*knockout in the previous study [19], one cannot exclude the possibility that Brpf3 plays a role in mESC function.

Myst2 is required for the maintenance of pluripotency and self-renewal in mESCs as described in our previous study [7], supporting the novel assertion that the fine-tuning of Myst2 protein levels by Brpf3 is important to the regulation of pluripotency and differentiation in ESCs. As our expectation, several differentiation-associated genes were dysregulated in Brpf3-overexpressing cells and furthermore, overexpression of Brpf3 failed to induce proper differentiation in EB formation and LIF withdrawal (Fig. 6). EB formation can represent many of the hallmarks of early embryonic development [20] and the cystic structure of EB mimics visceral endoderm-like cells [21]. Thus, developmental arrest by upregulation of Myst2 in Brpf3-overexpressing cells indicates that Brpf3-mediated regulation of Myst2 is critical for differentiation in mESCs. Collectively, our data provide insight into the unique and novel roles of Brpf3 regarding its functional interaction with Myst2 and resultant influence on protein stability, as well as its effects on differentiation in mESCs.

Recently, p53 and Myc were identified as the targets of E3 ligase Huwe1. Huwe1 ubiquitinates p53 in tumorigenesis [22] and also regulates Myc function by ubiquitination in tumor cell proliferation [22–25]. Furthermore, Huwe1 is involved in the control of neural differentiation and proliferation though ubiquitination of N-myc in mESCs [10]. Therefore, although our data indicate the inhibitory effect of Brpf3 on Huwe1-mediated ubiquitination of Myst2 (Fig. 3), it is necessary to investigate whether Brpf3 blocks Huwe1-mediated ubiquitination of other target proteins such as p53, N-Myc, and Miz1 in future studies.

In this study, the proteasomal regulation of Myst2 by Huwe1-Brpf3 was investigated in terms of pluripotency and differentiation of mESCs, but considerable evidence suggests a potential link between Huwe1-Brpf3-dependent regulation of Myst2 and other cellular processes. As mentioned before, both Huwe1 and Myst2 were reported to be involved in the regulation of DNA replication and DNA damage responses. Huwe1 was previously reported to be recruited at stalled replication forks [11] and to alleviate replication stress by interacting with PCNA [12] In addition, Huwe1 is linked to the regulation of the DNA damage response [13, 14]. Likewise, Myst2, as a key regulator of DNA replication licensing, activates replication origin firing via chromatin accessibility for the ORC complex by H3K14 acetylation [26–31]. Furthermore, Myst2 is known to be ubiquitinated under UV-damage conditions [9]. Moreover, Brpf3 is essential for H3K14 acetylation in replication origin activation and *Brpf3*-depleted cells endure replication stress-induced DNA damage efficiently [15]. In our data, we verified that Huwe1-Brpf3-Myst2 is new axis to control cell-cycle progression and DNA damage responses in mESCs (Fig. 7).

According to previous studies, while most cell types remain largely in G1-phase, ESCs have relatively high proportion of S-phase cells, which supports rapid progression of cell-cycle correlated with the accelerated cell proliferation in ESCs [32, 33]. Unusual cell-cycle progression of ESCs is also closely associated with differentiation. Differentiation induced by depletion of Oct4 causes increase of G1 phase and decrease of S phase in mESCs [34]. Inversely, dysregulation of cell-cycle progression by G1-cyclins knock-out leads to downregulation of core pluripotency-related genes such as Nanog, Oct4 and Sox2 and upregulation of differentiation-related genes, which induce differentiation of mESCs [35]. Thus, regulation of cell-cycle progression by Huwe1-Brpf3-Myst2 interrelationship is possible to influence proper differentiation.

In summary, we identified that Brpf3 regulates Myst2 protein stability by inhibiting the ubiquitination of Myst2. Interaction of Brpf3 with Myst2 is important for the control of Myst2 protein stability. Moreover, we determined that Huwe1 is a novel E3 ligase for Myst2 degradation in mESCs, and Brpf3 disturbs Huwe1-mediated ubiquitination of Myst2 via interaction with Huwe1 and Myst2. The expression profile of Huwe1 showed that inverse relation compared with those of Brpf3 and Myst2 during differentiation of mESCs. Thus, our data support the concept that Brpf3 and Huwe1 function antagonistically each other in regulation of Myst2 protein stability. More importantly, the fine-tuning of Myst2 protein stability by the Huwe1-Brpf3 partnership is not merely required to induce proper differentiation, but regulates cell-cycle progression in mESCs. This might be the first evidence showing the important role of Huwe1-Brpf3-Myst2 interrelationship in the early differentiation and cell-cycle progression of ESCs.

## Material and methods

### Cell cultures

E14Tg2a mouse ESCs, a kind gift from Youn, H. D (Seoul National University), were cultured on 0.1% gelatin-coated dishes under feeder-free conditions in KnockOut Dulbecco’s modified Eagle’s medium (Gibco/Thermo Fisher Scientific, Waltham, MA, USA) supplemented with 15% fetal bovine serum (Atlas Biologicals, Fort Collins, CO, USA), 2 mM L-glutamine, 100 U/mL penicillin, 100 µg/mL streptomycin, 100 µM 2-mercaptoethanol, 20 µg/mL ciprofloxacin, 1× non-essential amino acids, and 1000 U/mL leukemia inhibitory factor (LIF; MTI-GlobalStem, Gaithersburg, MD, USA). Stable E14Tg2a mouse ESCs with knockdown or overexpression of Brpf3 were cultured in medium supplemented with 2 µg/mL puromycin. 293T cells (ATCC) were cultured in Dulbecco’s modified Eagle’s medium (DMEM) supplemented with 10% fetal bovine serum, 100 U/mL penicillin, and 100 µg/mL streptomycin. All cell lines were tested for mycoplasma contamination.

### Establishment of knockdown and overexpression cell lines

To generate knockdown cell lines for Brpf3, shRNAs targeting the coding sequences and 3’ untranslated region (UTR) of Brpf3 mRNAs were cloned into a lentiviral-based pLKO.1 TRC cloning vector (Addgene plasmid 10879; kindly provided by Dr. David Root). Viral particles were produced as previously described [36]. For overexpression of Brpf3, E14tg2a cells were transfected with pCAG-FLAG-Brpf3 using DNA-In® Stem Transfection Reagent (Gibco/Thermo Fisher Scientific, Waltham, MA, USA). After 48 h, cells were selected by culturing in the presence of puromycin (1 μg/mL). For the construction of Brpf3-inducible overexpression cell lines, the Lenti-X Tet-On 3G inducible expression system was used following the manufacturer’s protocol (Cat#631353; Clontech/Takara Bio USA, Mountain View, CA, USA). Briefly, Brpf3 was cloned into the pLVX-TRE3G-ZsGreen1 vector. To produce viral particles, 293FT cells were transfected with 7 µg of pLVX-TRE3G vector containing Brpf3 or pLVX-Tet3G vector (regulator) with 2.25 µg of pMD2.G and 6.75 µg of psPAX2 using Lipofectamine™ 2000 transfection reagent (Invitrogen/Thermo Fisher Scientific). mESCs were first infected by viral particles containing pLVX-Tet3G vector harvested from 293FT cells and selected by culturing in the presence of G418 (600 ng/mL). Then, stable mESCs containing the regulator vector were infected with viral particles containing pLVX-TRE3G-Brpf3 and selected using puromycin (2 μg/mL). Expression of Brpf3 was induced by treatment with doxycycline.

### Construction of knock out cell lines using CRISPR-CAS9

To construct Brpf3 knock out cell lines, CRISPR-CAS9 system was used as previously described [6]. Briefly, we chose two target sgRNA sequences by searching on http://crispor.tefor.net/ and cloned sgRNA sequences into pX459 (Addgene plasmid 62988). After co-transfection of two CRISPR-CAS9 plasmids including sgRNAs, cells were selected by 2 µg/ml of puromycin. Homogeneous colonies grown from each single cell were harvested and checked its genomic mutation by Sanger sequencing. sgRNA sequences and PCR primer for Sanger sequencing are shown in Supplementary Table S1.

### Inhibitors

The treatment with BRPF family protein inhibitors was carried out as described previously [25, 37, 38]. To identify the effect of Brpf3 on Myst2 protein, we treated BRPF protein inhibitors to mESCs for 24 h: 5 µM of Brpf3 inhibitor NI-57 (Cayman), 10 µM of Brpf3 inhibitor OF-1 (Cayman), 1 µM of Brpf1 inhibitor PFI-4 (Cayman), and 5 µM of BAZ2A/B inhibitor BAZ-ICR2 (Cayman) as a negative control. To confirm the effect of Huwe1 on Myst2 protein stability, mESCs were treated with Huwe1 inhibitor BI8622 (MedChemExpress) for 24 h.

### Immunoprecipitation

Immunoprecipitation was performed as described previously [6]. Briefly, the cell lysates were incubated with anti-FLAG affinity gel (Sigma-Aldrich, St. Louis, MO, USA) or indicated antibodies overnight with rotation at 4 °C, followed by incubation with protein A beads for 4 h with rotation at 4 °C. The beads were washed five times with lysis buffer and then boiled with Laemmli SDS sample buffer (GenDEPOT, distributed by Thermo Fisher Scientific). Eluted protein complexes were analyzed by SDS-PAGE. To perform MS, FLAG-tagged Brpf3 expressed in 293T cells was purified with FLAG affinity gel and mixed with total protein extracts from mESCs.

For sequential immunoprecipitation, cell lysates were immunoprecipitated first using anti-FLAG affinity gel, followed by elution with 200 ng/µl 3XFLAG peptides by rotation for 30 min at 4 °C. Small part of eluted immunoprecipitates were boiled with Laemmli SDS sample buffer, and rest of them were immunoprecipitated again using Brpf3 antibody. After incubation with Brpf3 antibody, samples were incubated with protein A beads and then boiled with SDS sample buffer. Used antibodies are shown in Supplementary Table S2.

### In vivo ubiquitination assay

The ubiquitination of Myst2 in vivo was confirmed as previously described [39]. In short, cells were harvested after treatment 10 µM of MG132 (Sigma Aldrich) for 6 h and lysed by denaturing buffer (50 mM Hepes, pH 7.4, 150 mM NaCl, 1 mM EDTA, 2.5 mM MgCl_2_, 0.5% sodium deoxycholate, 1% Nonidet P-40, and 0.1% SDS) containing protease inhibitors and 10 mM deubiquitinase inhibitor N-ethylmaleimide (NEM). The cell lysates were immunoprecipitated by Myst2 antibody or anti-FLAG affinity gel for overnight at 4 °C, followed by incubation with protein A beads. The beads were washed five times with lysis buffer and boiled with Laemmli SDS sample buffer.

### Differentiation of mESCs

For spontaneous differentiation of mESCs, cells were cultured in LIF withdrawal medium for the indicated times in Fig. 6. Hanging drop culture was performed to form the embryoid bodies (EBs). Four thousand cells per 30 μL drop were pipetted onto the lids of Petri dishes for two days, then transferred to Petri dishes and incubated on an orbital shaker at 40 rpm for the indicated number of days in Fig. 6. The size of the EBs was measured using ImageJ software (NIH, Bethesda, MD, USA). The quantification of the EB sizes was performed using at least three independent experiments and more than 30 EBs were measured for each independent experiment. Data are presented as means ± SD.

### Commitment assay and alkaline phosphatase (AP) staining

As previously described [16], cell lineage commitment was induced by LIF withdrawal; after two days, cells were transferred and cultured for five days in LIF-containing medium. Cell differentiation patterns were detected via AP staining, using an AP detection kit (MilliporeSigma, Burlington, MA, USA) according to the manufacturer’s protocol. mESCs were stained with Fast Red Violet and Napthol AS-BI phosphate solutions for 15 min.

### RNA isolation and quantitative reverse transcription PCR (RT-qPCR)

RNA was extracted from mESCs using TRIReagent® (MRC, Cincinnati, OH, USA), then the extracted RNA was transcribed into cDNA using RevertAid First Strand cDNA Synthesis Kit (Thermo Fisher Scientific). For qPCR, SYBR® Premix Ex™ Taq II (Tli RNaseH Plus) and ROX Plus from Clontech/Takara Bio were used with an Applied Biosystems 7300 Real-time PCR system according to the manufacturers’ protocols. Used primer sequences are shown in Supplementary Table S3. The quantification of qRT-PCR data was performed using at least three independent experiments and data are presented as means ± SD.

### Immunostaining

Cells were cultured on glass slides coated with 0.1% gelatin then fixed with 4% paraformaldehyde and permeabilized by 0.2% Triton X-100 in phosphate-buffered saline (PBS). Cell incubation with primary and secondary antibodies was carried out as previously described [7], then visualized on an LSM 880 confocal microscope using ZEN Black software from Carl Zeiss AG (Oberkochen, Germany).

### Cell-cycle analysis by flow cytometry

For Bromodeoxyuridine (BrdU) incorporation assay, 1 mM of hydroxyurea (HU; Sigma-Aldrich, St. Louis, MO, USA) was treated for 24 h and 10 μM of BrdU (Sigma-Aldrich, St. Louis, MO, USA) was treated for 1 h before harvest. After fixation by cold 70% ethanol, DNA was hydrolyzed by HCl and neutralized by sodium borate subsequently. Using anti-BrdU FITC antibody (BD biosciences, San Jose, CA, USA), DNA incorporated with BrdU was stained. For cell-cycle profile analysis, cells were stained with 10 μg/ml propidium iodide containing RNase A for 30 min and then, analyzed by flow cytometry with fluorescence-activated cell sorting (FACS; LSRII, Beckman coulter). The quantification of FACS data was performed using at least three independent experiments and data are presented as means ± SD.

### SDS-PAGE and in-gel tryptic digestion for mass spectrometry (MS)

SDS-PAGE gel was sliced for in-gel tryptic digestion according to the manufacturer’s instructions using an in-gel tryptic digestion kit (Thermo Fisher Scientific, Rockford, IL). Briefly, the excised gels were destained, reduced by Tris[2-carboxyethyl] phosphine (TCEP), and alkylated by iodoacetamide (IAA). The alkylated gel pieces were dehydrated in 100% acetonitrile (ACN) and digested with MS grade trypsin in 25 mM NH_4_CO_3_ for 12 h at 30 ^o^C. Liquid present in the peptides was evaporated using a vacuum concentrator and cleaned-up using C18 spin columns (Thermo Fisher Scientific) for MS analysis.

### Liquid chromatography–mass spectrometry (LC-MS)/MS analysis and database search

The tryptic-digested peptides were analyzed on a Q Exactive™ Hybrid Quadrupole-Orbitrap™ mass spectrometer coupled with an Ultimate 3000 RSLCnano system (both from Thermo Fisher Scientific). The tryptic peptides were loaded onto trap columns (100 μm × 2 cm) packed with Acclaim™ PepMap™ 100 C18 resin (Thermo Fisher Scientific). The peptides were eluted with a linear gradient from 5 to 30% solvent B (0.1% formic acid in ACN) for 120 min at a flow rate of 300 nL/min. The eluted peptides separated using an analytical column (EASY-Spray™ column, 75 μm × 15 cm, Thermo Fisher Scientific) were sprayed into a nano-ESI source with an electrospray voltage of 2.4 kV. The Q Exactive™ Orbitrap™ mass analyzer was operated in a data-dependent (top 10) method. Full MS scans were acquired over a range of m/z 300–2000 with mass resolution of 70,000 (at m/z 200). The automatic gain control (AGC) target value was 1.00E + 06. The ten most intense peaks with charge state ≥2 were fragmented in the higher-energy collisional dissociation (HCD) collision cell with normalized collision energy of 25% and tandem mass spectra were acquired in the Orbitrap™ mass analyzer with a mass resolution of 17,500 at m/z 200.

Database searches for all raw data files were performed using Proteome Discoverer™ 2.2 software (Thermo Fisher Scientific). SEQUEST-HT (available with Proteome Discoverer™ software) was used to search against the Uniprot database. Database searching against the corresponding reversed database was also performed to evaluate the false discovery rate (FDR) of peptide identification. The database search parameters included up to two missed cleavages allowed for full tryptic digestion, precursor ion mass tolerance of 10 ppm, fragment ion mass tolerance of 0.02 Da, a fixed modification for carbamidomethyl cysteine, and variable modifications for methionine oxidation and N/Q deamination. We obtained an FDR of less than 1% on the peptide level, filtered with high peptide confidence.

### Statistical analysis

Data are expressed as means ± SD (*n* = 3). Pairwise comparisons were performed using two-tailed Student’s *t* tests or one-way ANOVA. Symbols used to indicate significant differences are as follows: *p < 0.05, **p < 0.01, and ***p < 0.001. We were not blinded to the group allocation during the experiment.

## Supplementary information

Supplementary Table S1

Supplementary Table S2

Supplementary Table S3

Supplementary Figure legends

Supplementary Figure S1

Supplementary Figure S2

Supplementary Figure S3

Supplementary Figure S4

Supplementary Figure S5

Supplementary Figure S6

Supplementary Figure S7

## References

[1] Klein BJ, Lalonde ME, Côté J, Yang XJ, Kutateladze TG (2014). Crosstalk between epigenetic readers regulates the MOZ/MORF HAT complexes. Epigenetics..

[2] Hibiya K, Katsumoto T, Kondo T, Kitabayashi I, Kudo A (2009). Brpf1, a subunit of the MOZ histone acetyl transferase complex, maintains expression of anterior and posterior Hox genes for proper patterning of craniofacial and caudal skeletons. Dev Biol..

[3] You L, Li L, Zou J, Yan K, Belle J, Nijnik A (2016). BRPF1 is essential for development of fetal hematopoietic stem cells. J Clin Invest.

[4] You L, Yan K, Zhou J, Zhao H, Bertos NR, Park M (2015). The lysine acetyltransferase activator Brpf1 governs dentate gyrus development through neural stem cells and progenitors. PLoS Genet..

[5] Mishima Y, Miyagi S, Saraya A, Negishi M, Endoh M, Endo TA (2011). The Hbo1-Brd1/Brpf2 complex is responsible for global acetylation of H3K14 and required for fetal liver erythropoiesis. Blood..

[6] Cho HI, Kim MS, Jang YK (2016). The BRPF2/BRD1-MOZ complex is involved in retinoic acid-induced differentiation of embryonic stem cells. Exp Cell Res.

[7] Kim MS, Cho HI, Park SH, Kim JH, Chai YG, Jang YK (2015). The histone acetyltransferase Myst2 regulates Nanog expression, and is involved in maintaining pluripotency and self-renewal of embryonic stem cells. FEBS Lett.

[8] Zou C, Chen Y, Smith RM, Snavely C, Li J, Coon TA (2013). SCFFbxw15mediates histone acetyltransferase binding to origin recognition complex (HBO1) ubiquitin-proteasomal degradation to regulate cell proliferation. J Biol Chem.

[9] Matsunuma R, Niida H, Ohhata T, Kitagawa K, Sakai S, Uchida C (2016). UV damage-induced phosphorylation of HBO1 triggers CRL4. Mol Cell Biol.

[10] Zhao X, Heng JIT, Guardavaccaro D, Jiang R, Pagano M, Guillemot F (2008). The HECT-domain ubiquitin ligase Huwe1 controls neural differentiation and proliferation by destabilizing the N-Myc oncoprotein. Nat Cell Biol.

[11] Coleman KE, Huang TT (2011). Replication forks. EMBO Rep..

[12] Choe KN, Nicolae CM, Constantin D, Imamura Kawasawa Y, Delgado‐Diaz MR, De S (2016). HUWE1 interacts with PCNA to alleviate replication stress. EMBO Rep..

[13] Fok KL, Bose R, Sheng K, Chang CW, Katz-Egorov M, Culty M (2017). Huwe1 regulates the establishment and maintenance of spermatogonia by suppressing DNA damage response. Endocrinology..

[14] Mandemaker IK, Van Cuijk L, Janssens RC, Lans H, Bezstarosti K, Hoeijmakers JH (2017). DNA damage-induced histone H1 ubiquitylation is mediated by HUWE1 and stimulates the RNF8-RNF168 pathway. Sci Rep..

[15] Feng Y, Vlassis A, Roques C, Lalonde M-E, Gonzalez-Aguilera C, Lambert J-P (2016). BRPF3-HBO1 regulates replication origin activation and histone H3K14 acetylation. EMBO J..

[16] Zhou X, Smith AJH, Waterhouse A, Blin G, Malaguti M, Lin CY (2013). Hes1 desynchronizes differentiation of pluripotent cells by modulating STAT3 activity. Stem Cells..

[17] Doyon Y, Cayrou C, Ullah M, Landry AJ, Côté V, Selleck W (2006). ING tumor suppressor proteins are critical regulators of chromatin acetylation required for genome expression and perpetuation. Mol Cell.

[18] Choi EH, Yoon S, Park KS, Kim KP (2017). The homologous recombination machinery orchestrates post-replication DNA repair during self-renewal of mouse embryonic stem cells. Sci Rep..

[19] Yan K, You L, Degerny C, Ghorbani M, Liu X, Chen L (2016). The chromatin regulator BRPF3 preferentially activates the HBO1 acetyltransferase but is dispensable for mouse development and survival. J Biol Chem.

[20] Bratt-Leal AM, Carpenedo RL, McDevitt TC (2009). Engineering the embryoid body microenvironment to direct embryonic stem cell differentiation. Biotechnol Prog..

[21] Sugimoto M, Kondo M, Hirose M, Suzuki M, Mekada K, Abe T (2012). Molecular Identification of tw5: Vps52 promotes pluripotential cell differentiation through cell-cell interactions. Cell Rep.

[22] Chen D, Kon N, Li M, Zhang W, Qin J, Gu W (2005). ARF-BP1/mule is a critical mediator of the ARF tumor suppressor. Cell..

[23] Jin J, Liu J, Chen C, Liu Z, Jiang C, Chu H (2016). The deubiquitinase USP21 maintains the stemness of mouse embryonic stem cells via stabilization of Nanog. Nat Commun..

[24] Adhikary S, Marinoni F, Hock A, Hulleman E, Popov N, Beier R (2005). The ubiquitin ligase HectH9 regulates transcriptional activation by Myc and is essential for tumor cell proliferation. Cell..

[25] Peter S, Bultinck J, Myant K, Jaenicke LA, Walz S, Muller J (2014). Tumor cell-specific inhibition of MYC function using small molecule inhibitors of the HUWE1 ubiquitin ligase. EMBO Mol Med.

[26] Miotto B, Struhl K (2010). HBO1 histone acetylase activity is essential for DNA replication licensing and inhibited by geminin. Mol Cell..

[27] Burke TW, Cook JG, Asano M, Nevins JR (2001). Replication factors MCM2 and ORC1 interact with the histone acetyltransferase HBO1. J Biol Chem.

[28] Chadha GS, Blow JJ (2010). Histone acetylation by HBO1 tightens replication licensing. Mol Cell.

[29] Wong PG, Glozak MA, Cao TV, Vaziri C, Seto E, Alexandrow MG (2010). Chromatin unfolding by Cdt1 regulates MCM loading via opposing functions of HBO1 and HDAC11-geminin. Cell Cycle..

[30] Miotto B, Struhl K (2008). HBO1 histone acetylase is a coactivator of the replication licensing factor Cdt1. Genes Dev..

[31] Iizuka M, Matsui T, Takisawa H, Smith MM (2006). Regulation of replication licensing by acetyltransferase Hbo1. Mol Cell Biol.

[32] Roccio M, Schmitter D, Knobloch M, Okawa Y, Sage D, Lutolf MP (2013). Predicting stem cell fate changes by differential cell cycle progression patterns. Development..

[33] White J, Dalton S (2005). Cell cycle control of embryonic stem cells cell cycle fundamentals. Stem Cell Rev.

[34] Lee J, Go Y, Kang I, Han Y-M, Kim J (2009). Oct-4 controls cell-cycle progression of embryonic stem cells. Biochem J.

[35] Liu L, Michowski W, Inuzuka H, Shimizu K, Nihira NT, Chick JM (2017). G1 cyclins link proliferation, pluripotency and differentiation of embryonic stem cells. Nat Cell Biol.

[36] Lee J, Kim MS, Park SH, Jang YK (2018). Tousled-like kinase 1 is a negative regulator of core transcription factors in murine embryonic stem cells. Sci Rep..

[37] Wu Q, Heidenreich D, Zhou S, Ackloo S, Krämer A, Nakka K (2019). A chemical toolbox for the study of bromodomains and epigenetic signaling. Nat Commun.

[38] Lima-Fernandes E, Murison A, da Silva Medina T, Wang Y, Ma A, Leung C (2019). Targeting bivalency de-represses Indian Hedgehog and inhibits self-renewal of colorectal cancer-initiating cells. Nat Commun..

[39] Park A, Ra EA, Lee TA, Choi H, Lee E, Kang S (2019). HCMV-encoded US7 and US8 act as antagonists of innate immunity by distinctively targeting TLR-signaling pathways. Nat Commun..

