# Supplementary material for: BRPF3-HUWE1-mediated regulation of MYST2 is required for differentiation and cell-cycle progression in embryonic stem cells
Source: Cell Death Differ. 2020 Jun 18;27(12):3273–88. doi: 10.1038/s41418-020-0577-1 (PMC7853152; doi:10.1038/s41418-020-0577-1)
Supplement: Supplementary file 1 — Supplementary Table S1 [file 41418_2020_577_MOESM1_ESM.docx]

Supplementary Table S1. sgRNA sequences targeting Brpf3 and PCR primers for confirmation

| Oligonucleotides | SOURCE | IDENTIFIER |
| --- | --- | --- |
| CAS-Brpf3-1 forward : CACCGATTGTTGAGGTCGACATCGA | This paper | N/A |
| CAS-Brpf3-1 reverse : AAACTCGATGTCGACCTCAACAATC | This paper | N/A |
| CAS-Brpf3-2 forward : CACCTGGGTCTTGACCCGTTTTAT | This paper | N/A |
| CAS-Brpf3-2 reverse : AAACATAAAACGGGTCAAGACCCAC | This paper | N/A |
| CAS-Brpf3-Confirm primer forward :  ACAGGATCCTCCACCTGTCTCTGCCTCTT | This paper | N/A |
| CAS-Brpf3-Confirm primer reverse :  GCGAAGCTTGGTAATATCCTGAGCAGTCAGC | This paper | N/A |
