# Supplementary material for: BRPF3-HUWE1-mediated regulation of MYST2 is required for differentiation and cell-cycle progression in embryonic stem cells
Source: Cell Death Differ. 2020 Jun 18;27(12):3273–88. doi: 10.1038/s41418-020-0577-1 (PMC7853152; doi:10.1038/s41418-020-0577-1)
Supplement: Supplementary file 2 — Supplementary Table S2 [file 41418_2020_577_MOESM2_ESM.docx]

Supplementary Table S2. Antibodies list

| Antibodies | SOURCE | IDENTIFIER |
| --- | --- | --- |
| Mouse monoclonal anti-FLAG | Sigma Aldrich | F3165 |
| Rabbit polyclonal anti-FLAG | Sigma Aldrich | F7425 |
| Rabbit polyclonal anti-KAT7/Hbo1/MYST2 | abcam | Ab70183 |
| Rabbit polyclonal anti-alpha tubulin | Ab frontier | LF-PA0146 |
| Rabbit polyclonal anti-Brpf3 | BETHYL | A304-082A |
| Mouse monoclonal anti-HA | abcam | Ab18181 |
| Rabbit polyclonal anti-Huwe1 | abcam | Ab70161 |
| Goat anti-Rabbit IgG (H+L) Cross-Adsorbed Secondary Antibody, Alexa Fluor 568 | Invitrogen | A11011 |
| Goat Anti-Rabbit IgG H&L (HRP) | abcam | Ab6721 |
| Goat Anti-Mouse IgG H&L (HRP) | abcam | Ab6789 |
