# Supplementary material for: BRPF3-HUWE1-mediated regulation of MYST2 is required for differentiation and cell-cycle progression in embryonic stem cells
Source: Cell Death Differ. 2020 Jun 18;27(12):3273–88. doi: 10.1038/s41418-020-0577-1 (PMC7853152; doi:10.1038/s41418-020-0577-1)
Supplement: Supplementary file 3 — Supplementary Table S3 [file 41418_2020_577_MOESM3_ESM.docx]

Supplementary Table S3. Oligonucleotides list

| Oligonucleotides | SOURCE | IDENTIFIER |
| --- | --- | --- |
| Primer for qRT-PCR |  |  |
| Mouse Bmp4 forward : AGGAGGAGGAAGAGCAGA | This paper | N/A |
| Mouse Bmp4 reverse : TGGGATGTTCTCCAGATG | This paper | N/A |
| Mouse T forward : ACACGGCTGTGAGAGGTA | This paper | N/A |
| Mouse T reverse : ATGACTCACAGGCAGCAT | This paper | N/A |
| Mouse Nanog forward : TGCACTCAAGGACAGGTT | This paper | N/A |
| Mouse Nanog reverse : TGCACTTCATCCTTTGGT | This paper | N/A |
| Mouse Gata4 forward : ATCAAACCCCTTGCTCTC | This paper | N/A |
| Mouse Gata4 reverse : AGGTGCAGATGAGCCATA | This paper | N/A |
| Mouse Flk1 forward : TTCATCGCCTCTGTCAGT | This paper | N/A |
| Mouse Flk1 reverse : CTGGATACCTAGCGCAAA | This paper | N/A |
| Mouse Brpf3 forward : AACGGCTTTGGAAAACACAC | This paper | N/A |
| Mouse Brpf3 reverse : TCCTCCACTGAGACCCAAAC | This paper | N/A |
| Mouse Myst2 forward : TCAGGATGCCCACTGTATCA | This paper | N/A |
| Mouse Myst2 reverse : TGTGCTCTCACCTTGCATTC | This paper | N/A |
| Mouse Gapdh forward : TGGCAAAGTGGAGATTGT | This paper | N/A |
| Mouse Gapdh reverse : TCCTGGAAGATGGTGATG | This paper | N/A |
| shRNA for Brpf3 (CDS-1) : GAGGTCTGCTTCGCTAATAC | This paper | N/A |
| shRNA for Brpf3 (CDS-2) : GTTCGCAAGACTGCGTATTG | This paper | N/A |
| shRNA for Brpf3 (CDS-3) : CTGCGACATCTGCAACTTAG | This paper | N/A |
| shRNA for Brpf3 (3’UTR) : CCGAGGGCAAGACCCAATATT | This paper | N/A |
| shRNA for Luciferase : AGAGCTGTTTCTGAGGAGCCT | This paper | N/A |
