# Supplementary material for: BRPF3-HUWE1-mediated regulation of MYST2 is required for differentiation and cell-cycle progression in embryonic stem cells
Source: Cell Death Differ. 2020 Jun 18;27(12):3273–88. doi: 10.1038/s41418-020-0577-1 (PMC7853152; doi:10.1038/s41418-020-0577-1)
Supplement: Supplementary file 4 — Supplementary Figure legends [file 41418_2020_577_MOESM4_ESM.docx]

**Supplementary Figure S1**. (A) mRNA level of Brpf3 and Myst2 in Brpf3 overexpressing mESCs. mRNA expression level of Brpf3 and Myst2 was analyzed by RT-qPCR in control (pCAG) and Brpf3-overexpressed mESCs (pCAG-Brpf3). The mRNA expression levels were normalized to that of GAPDH. The expression levels of the control were set at 1. (n=3). *p < 0.05, **p < 0.01, ***p < 0.001, compared with control. (B) Representative PCR genotyping result from E14tg2A (control) and *Brpf3*^(+/-)^. Note a single band (703 bp) in control and two bands (703 bp and 224 bp) in *Brpf3*^(+/-)^. (C) The Sanger sequencing of PCR products from (B). Knock-out allele of *Brpf3*^(+/-)^ cell lines was purified and analyzed.

**Supplementary Figure S2.** (A) Protein level of Myst2 after treatment with 10 ug/mL cycloheximide for indicated times was investigated by western blot analysis. Alpha-tubulin was used as the loading control. (B) Protein level of Myst2 after treatment with 10 μM MG132 for six hours was analyzed by western blot. Alpha-tubulin was used as the loading control. (C) Ubiquitination of Myst2 was detected by immunoprecipitation using anti-FLAG affinity gel. pCAG-FLAG and pCAG-FLAG-Myst2 were co-transfected with HA-Ubiquitin in E14tg2a cells and treated with 10 μM of MG132 for six hours. Alpha-tubulin was used as the loading control.

**Supplementary Figure S3.** Interaction of the Huwe1-Brpf3-Myst2 complex was analyzed by co-immunoprecipitation using an anti-FLAG affinity gel. Co-immunoprecipitation was performed in pCAG-FLAG-Myst2, pCAG-FLAG-Brpf3, or pCAG-FLAG-Huwe1 overexpressed 293T cells. Immunoprecipitates were analyzed by western blot with anti-FLAG, anti-Myst2, anti-Brpf3, and Huwe1 antibodies.

**Supplementary Figure S4**. (A) Schematic diagram of wild-type and deletion forms of Brpf3. (B) Co-immunoprecipitation of Myst2 with FLAG-tagged wild-type (WT) and deletion mutants of Brpf3 (N127, ΔN127) was performed with anti-FLAG affinity gel. Immunoprecipitates were analyzed by western blot with anti-FLAG and anti-Myst2 antibodies. Alpha-tubulin was used as the loading control. (C) Protein level of Myst2 in over-expression of wild-type (WT) and deletion forms of Brpf3 (N127 and ΔN127) cell lines was investigated by western blot analysis. Alpha-tubulin was used as the loading control. (D) Protein level of Myst2 in overexpression of wild-type (WT) and deletion mutants of Brpf3 (N127, ΔN127, ΔPWWP, PWWP) cell lines was investigated by western blot analysis. Alpha-tubulin was used as the loading control. Asterisk symbol(s) indicates the protein band(s) which was expressed from each FLAG-tagged Brpf3 construct.

**Supplementary Figure S5.** Interaction of Huwe1 with FLAG-tagged wild-type (WT) and deletion forms of Brpf3 (N127, ΔN127) was confirmed by immunoprecipitation with anti-FLAG affinity gel. Immunoprecipitates were analyzed by western blot with anti-FLAG and anti-Huwe1 antibodies.

**Supplementary Figure S6.** (A) Embryoid body (EB) formation ability in control (shLuc) and Myst2-knockdowned cell lines was investigated using the hanging drop assay. Phase-contrast images of EB at two-days after EB formation (left panel) and quantification of the size of EBs (n=3; right panel). More than 30 EBs were measured for each independent experiment. The size of EB control was set at 1. **p* < 0.05, ***p* < 0.01, ****p* < 0.001, compared with control. Scale bar: 1 mm (B) Cyst formation in EB was observed in control (shLuc) and Myst2-knockdowned cell lines. Phase-contrast images of EB at six-days after EB formation. Red arrows indicate cystic structures of EB. Scale bar: 1 mm (C) Morphology of control (shLuc) and Myst2-knockdowned cell lines after induction of differentiation by LIF withdrawal was investigated by phase-contrast images and alkaline phosphatase staining. Scale bar: 0.5 mm

**Supplementary Figure S7.** (A) Bar graph representing relative cell populations in cell-cycle phases G1, S, and G_2_/M in Myst2 overexpressed mESCs released for 5 hours after double thymidine block. (B) Bar graph representing relative cell populations in cell-cycle phases G1, S, and G_2_/M in Myst2-knockdowned mESCs released for 5 hours after double thymidine block. *p < 0.05, **p < 0.01, ***p < 0.001, compared with control cells.
